# Plastic Operations on the Palate

**Published:** 1885-03

**Authors:** 


### Plastic Operations on the Palate.

M. Ulysse Trelat, in a paper on plastic operations on the palate, and the determination of the age at which it is proper to operate, reaches the following conclusions:

1. It is not necessary to perform plastic operations in this locality before the child has reached its seventh year.
2. Children who are to be operated upon should be carefully drilled in the articulation of vocal sounds from the time they first begin to speak up to the time of operation. Functional cure is hastened and the ability to enunciate distinctly is rendered more certain by such a course.

Should harelip exist along with cleft palate, the indications for operation are no longer the same. In such cases it is advisable to operate in the first month or within six months after birth. The measure of success, as relates to ability to articulate distinctly, here again depends upon careful training before and after the operation.

M. Trelat further remarks concerning the operation, that when the division extends from the alveolar border to the veil of the palate and palatine arch, success is possible, but failure often results. Plastic operation is unnecessary when the fissure is not of sufficient width to admit fragments of considerable size. Simply bringing and retaining the parts in apposition is preferable.

Plastic operations are not dangerous. In forty-six cases operated upon by M. Trelat, all recovered. He operates with the patient in a horizontal position, the head bent backwards, so as to prevent as fully as possible, access of blood to the bronchi. The food should be semi-liquid for ten or twelve days succeeding the operation. Cure ordinarily is effected in from four to eight days. —*Medical News.*

